# Investigation of the Approaches to Optimal Exercise Interventions Based on Dementia Type: A Theoretical Review

**DOI:** 10.3390/healthcare12050576

**Published:** 2024-03-01

**Authors:** Hyun Lee, Hyeongmin Lee, Jinhyung Choi, Gyujeong Hwang, Hyemin Lee, Hyunmin Lee, Sujeong Kim, Minjeong Kim, Huiju Nam, Jaeyoon Shim, Hatem Jaber, Jongeun Yim

**Affiliations:** 1 Department of Physical Therapy, Graduate School of Sahmyook University, Seoul 01795, Republic of Korea; lh5135@syuin.ac.kr (H.L.); gudals1098@syuin.ac.kr (H.L.); cjh_ssam@syuin.ac.kr (J.C.); phdhgj@syuin.ac.kr (G.H.); lhm031@syu.ac.kr (H.L.); happyday@syuin.ac.kr (H.L.); tnwjd5660@syuin.ac.kr (S.K.); anyminj@syuin.ac.kr (M.K.); nhj1396@syuin.ac.kr (H.N.); dinojae@syuin.ac.kr (J.S.); 2 College of Rehabilitative Sciences, University of St. Augustine for Health Sciences, Austin, TX 78739, USA; hjaber@usa.edu

**Keywords:** dementia, cognitive dysfunction, physical examination, exercise therapy

## Abstract

The aim of this study is to enhance comprehension of the different types and features of dementia, including their symptoms, diagnosis and medical treatment, and to propose various evidence-based exercise interventions and their clinical applications tailored to each specific type of dementia. The theoretical review includes the analysis of publications in the scientific databases PubMed/Medline, Ebsco, Scielo, and Google. A total of 177 articles were found, of which 84 were studied in depth. With the prevalence of all forms of dementia projected to increase from 57.4 million in 2019 to 152.8 million in 2050, personalized treatment strategies are needed. This review discusses various forms of dementia, including their pathologies, diagnostic criteria, and prevalence rates. The importance of accurate diagnosis and tailored care is emphasized, as well as the effectiveness of physical exercise in improving cognitive function in dementia patients. For Alzheimer’s, a combination of drug therapies and exercises is recommended to enhance cerebral blood flow and neurotransmitter activity. To improve cognitive and motor functions in Lewy body dementia, a combination of pharmacological and physical therapies is recommended. For managing frontotemporal dementia, a mix of medication and exercises aimed at emotion regulation, including aerobic exercises, and a unified protocol, is suggested. For mild cognitive impairment, aerobic and functional exercises are important in delaying cognitive decline and enhancing cognitive performance. In conclusion, individualized care and treatment plans tailored to the specific characteristics of each disease type can improve the quality of life for individuals with this condition and effectively manage this growing global health issue.

## 1. Introduction

The global population of individuals aged 60 years and older is estimated to be approximately two billion by 2050, and rising life expectancy has contributed to a rapid increase in this demographic. This increase is associated with a higher prevalence of chronic conditions including dementia [1]. Based on the Global Burden of Disease (GBD) study, the estimated number of individuals with dementia was approximately 57.4 million in 2019 and this number is projected to increase significantly to 152.8 million by 2050. This estimated increase indicates that as the population affected by dementia continues to grow, the demand for public health initiatives and policies to address this issue effectively will increase [2].

Dementia is a neurodegenerative disorder characterized by memory loss, impaired cognitive ability, and reduced functional capacity to perform daily activities. Primary underlying pathologies include Alzheimer’s disease, vascular dementia, Lewy body dementia, and frontotemporal dementia. Other conditions, such as normal pressure hydrocephalus (NPH) and mild cognitive impairment (MCI) may also be involved. Statistics have revealed that Alzheimer’s disease accounts for approximately 60% of all dementia cases, whereas vascular dementia accounts for approximately 20%. Differentiating between Alzheimer’s disease and vascular dementia can be challenging because of overlapping symptoms, underlying mechanisms, and risk factors [3]. Each pathology contributes to the overall disease spectrum to varying degrees, resulting in a diverse range of patients, including those with pure cerebrovascular disease and Alzheimer’s disease, which represent the extreme ends of the spectrum [4].

Age is a crucial factor in the prevalence of dementia, as it is strongly associated with the risk of developing dementia. Recent studies have indicated a decline in the age-adjusted incidence of dementia in the United States and other developed countries over the past two decades, which is believed to be linked to a higher level of formal education [5]. Age-adjusted dementia refers to the adjustment of the statistical prevalence of dementia to account for variations in age distribution of the population. The decrease in age-adjusted dementia indicates that an improved understanding of the disease, early diagnosis, and prompt treatment can help reduce its occurrence [6]. Therefore, accurate diagnosis and early intervention for each type of dementia are crucial, and diagnostic methods tailored to the characteristics of each form of dementia are needed.

There are two primary treatment approaches for dementia: pharmacological and nonpharmacological. Both pharmacological and nonpharmacological treatments are crucial for achieving treatment goals in individuals with dementia. However, the significance of nonpharmacological treatments has been increasingly highlighted owing to the potential side effects associated with continuous medication use. In one study, Shin [7] explored the benefits of exercise as a non-drug treatment option. In a study comparing two groups of elderly individuals, one of which engaged in aerobic exercise and the other in non-aerobic strength training, positive exercise effects were observed in the group that engaged in aerobic exercise after six months. Specifically, the anterior white matter significantly increased in volume, as did the frontal regions, such as the anterior cingulate gyrus, supplementary motor cortex, and right inferior frontal gyrus, compared to the non-aerobic group [8]. After the frontal lobe, the temporal lobe, which is associated with the hippocampus, is the region most significantly affected by aging [9]. Erickson et al. [10] revealed that aerobic exercise induces structural changes in the hippocampus, indicating that it is more effective than non-aerobic strength training in the general population. However, these findings are inconsistent when specifically targeting individuals with cognitive impairment. A meta-analysis conducted on cognitively impaired patients aged 50 years and above showed that physical exercise interventions improved cognitive function in older adults, regardless of their baseline cognitive status. The study found that interventions involving aerobic exercise, resistance training, combined training, and tai chi were equally effective [11]. The proven efficacy of various types of exercises suggests that they can be integrated into the daily routines of individuals with dementia. Unlike medications, which may have side effects, physical exercise is not associated with adverse effects and is a cost-effective treatment option. The novelty of the study lies in its comprehensive and tailored approach towards non-pharmacological interventions for different types of dementia. This theoretical review uniquely focuses on the individual characteristics of various dementia types, including Alzheimer’s Disease, Lewy Body Dementia, Frontotemporal Dementia, and Mild Cognitive Impairment, and proposes evidence-based, non-pharmacological interventions specifically suited to each type. This individualized approach is particularly innovative, as it diverges from the more general strategies often seen in dementia care. Further studies should be conducted to investigate the therapeutic potential of physical exercise. However, with the exception of Alzheimer’s disease, which has the highest prevalence, exercise interventions for other types of dementia have not been studied extensively. Hence, additional research is warranted to determine which exercises are most effective for each type of dementia.

Therefore, this theoretical review aims to gain a deeper understanding of the various types and features of dementia, including their diagnostic methods, and to propose the most effective exercise interventions tailored to each specific type of dementia.

## 2. Methods

A Theoretical Review was carried out as part of a research project to determine the current state of knowledge on the subject under study. The Theoretical Review contemplates the analysis of publications on dementia, including symptoms, diagnosis and interventions (pharmacological and non-pharmacological), published in the period from 2000 to 2023, in the scientific databases PubMed/Medline, Ebsco, Scielo, and Google Scholar, considering clinical cases, review articles, and clinical trials using the search terms “dementia”, “Alzheimer’s disease”, “Lewy body dementia”, “frontotemporal dementia”, and “mild cognitive impairment” along with “symptom”, “diagnosis”, “intervention”, “non-pharmacological”, and “exercise”. A total of 177 articles were found, of which 84 were studied in depth. Three papers published before 2000 were included in this review due to their importance to their substantive field as demonstrated by extensive citations in other papers.

## 3. Types and Symptoms of Dementia

### 3.1. Alzheimer’s Disease

Alzheimer’s disease (AD) is the most common form of dementia. It is characterized by the accumulation of amyloid-beta (Aβ) protein in the brain and reduced concentration of brain-derived neurotrophic factor (BDNF). These changes cause neuronal damage in the hippocampus, leading to the gradual degeneration and death of brain nerve cells, which in turn results in cognitive decline in learning, memory, and language [12]. In brain histology, characteristic lesions are observed, including senile plaques and neurofibrillary tangles formed by tau protein hyperphosphorylation due to Aβ protein deposition, and brain atrophy develops due to neuronal loss. Senile plaques tend to accumulate in the temporal and parietal lobes, which are responsible for memory and language. The accumulation of these substances is associated with the expression of dementia symptoms [13].

Under normal circumstances, small amounts of Aβ protein are produced to protect nerve cells from inflammatory substances. These proteins are quickly degraded by the spinal fluid and dispersed in the cerebrospinal fluid without any specific structure [4]. However, when abnormally high amounts of Aβ protein are produced, it is not broken down, and accumulates around nerve cells, ultimately leading to plaque formation. Under normal conditions, tau proteins maintain the structure and stability of nerve cells, and contribute to intracellular nutrient transport. However, abnormally folded tau proteins aggregate and are difficult to degrade, leading to the formation of neurofibrillary tangles within neurons, resulting in neuronal dysfunction and cell death [13].

### 3.2. Lewy Body Dementia

Lewy body dementia (LBD) is the third most prevalent type of dementia after Alzheimer’s disease and vascular dementia, accounting for 15–24% of individuals diagnosed with dementia in clinical settings [14]. In 1912, Lewy first described cytoplasmic inclusions, known as Lewy bodies, in Parkinson’s disease (PD) [15]. Cortical Lewy bodies were first reported in association with dementia in 1961 (Okazaki H) but remained relatively uncommon until the 1980s. However, the ease of identifying Lewy bodies through ubiquitin and α-synuclein immunostaining led to the demonstration that Lewy bodies are the second most common neuropathological finding in dementia, after Alzheimer’s disease [16]. It is currently incurable and is thought to be caused by the accumulation of ubiquitin and α-synuclein proteins, which are commonly found in the substantia nigra of the brainstem. When Lewy bodies become widespread throughout the brain, they cause dementia symptoms similar to those observed in AD. However, there are differences between LBD and AD [17]. Typical symptoms of LBD include impaired memory, attention, and executive functioning; episodic hallucinations and delusions; PD symptoms; loss of consciousness; and disturbances in eye movement, behavior, and sleep [18]. In contrast, in Alzheimer’s, a gradual decline in memory and memory loss are prominent in the early stages, whereas neuropsychiatric symptoms usually appear in later stages [19]. The prognosis of Lewy body dementia is poor, with a life expectancy of 5–8 years. In addition, the causes of death vary and are complex, including malnutrition, falls, dysphagia, and pneumonia [17].

### 3.3. Frontotemporal Dementia

Frontotemporal dementia (FTD) is a severe neurological condition characterized by a gradual decline in behavior, executive function, and language abilities. It is the third most prevalent form of dementia across all age groups after Alzheimer’s disease and Lewy body dementia. Additionally, FTD is one of the primary causes of early onset dementia, which occurs at a relatively young age [20]. Clinically, the Frontotemporal Lobar Degeneration (FTLD) spectrum can be divided into three FTD syndromes based on distinct early characteristics. The behavioral variant of FTD (bvFTD) is characterized by a gradual decline in behavioral and executive functions, primarily affecting the frontal lobe. Semantic dementia (SD) is characterized by loss of semantic knowledge accompanied by atrophy of the left temporal lobe, which is more pronounced than that of the right frontal lobe. Finally, progressive non-fluent aphasia (PNFA), characterized by deficits in language expression or motor skills, is predominantly associated with atrophy of the left perisylvian region. The clinical presentation, pathology, and underlying genetics considerably overlap between FTD and other conditions, including motor neuron disease/amyotrophic lateral sclerosis, atypical Parkinson’s syndrome, progressive supranuclear palsy, and corticobasal syndrome. Although all clinical FTD syndromes may be associated with motor neuron diseases, these associations are most commonly observed in bvFTD [21].

The exact mechanisms underlying FTD development are poorly understood. However, advances in molecular biology and immunohistochemical staining techniques have enabled the classification of FTLD spectrum disorders into three main categories based on the primary neuropathological proteins involved: (i) microtubule-associated protein tau (FTLD-TAU), (ii) TAR DNA-binding protein-43 (FTLD-TDP), and (iii) fusion proteins within sarcomeres (FTLD-FUS). As the disease progresses, the various clinical variants of FTD converge due to underlying neuropathological changes and patterns of atrophy. This eventually affects a larger extent of the frontal and temporal lobes. The clinical presentation of FTD can initially manifest as abnormalities in personality, behavior, or language, which later progress to a more generalized cognitive decline. This adds to the complexity and heterogeneity of accurately diagnosing FTD and predicting its neuropathological prognosis [22].

### 3.4. Mild Cognitive Impairment

Mild Cognitive Impairment (MCI) is defined as a level of cognitive impairment greater than what is expected for an individual’s age and education level in one or more cognitive domains. However, unlike dementia, it does not significantly interfere with daily functioning. The prevalence rates of MCI among adults aged 65 years and older range from 3% to 19%, indicating substantial variations [23]. Gauthier et al. noted that some individuals with MCI appeared to remain stable or even return to normal over time [24]. However, more than half of the individuals with MCI progress to dementia within five years, and MCI is particularly associated with a higher risk of progression to Alzheimer’s disease, a subtype of memory loss. It should be noted, therefore, that MCI and dementia (Alzheimer’s disease) are distinct concepts, necessitating the need for more accurate diagnostic methods [25].

The diagnostic criteria for MCI currently require objective evidence of cognitive impairment in one or more cognitive domains, such as memory, executive function, attention, language, or visuospatial skills, without significant impairment in social or occupational functioning [26]. Studies involving older adults with MCI have reported that physical exercise positively affects the overall cognitive function, executive function, attention, and delayed memory [27]. However, research on older adults with dementia has shown that physical exercise does not affect cognition [28]. Therefore, it is important to conduct various assessments to differentiate between MCI and dementia. These assessments should include cognitive function tests, functional status assessments, medication use, and evaluation of neurological or psychiatric abnormalities [29].

## 4. Diagnosis and Physical Examination of Dementia

### 4.1. Alzheimer’s Disease

Alzheimer’s disease places a significant burden on the quality of life of patients and their caregivers, including social and economic challenges. Previous research has demonstrated that early diagnosis and treatment can significantly impact a patient’s disease progression [30]. The Mini-Mental State Examination (MMSE) is a widely used diagnostic tool for dementia. The questionnaire used in this study was standardized by Folstein et al. A score of 17 or less indicates severe cognitive decline, 18–23 indicates mild cognitive decline, and 24 or more indicates normal cognitive function [31]. The Clinical Dementia Rating Scale (CDR) is a rating scale developed by Morris that assesses the overall cognitive function in dementia. This scale measures six domains: memory, perception, judgment and problem-solving, community living, residential living, hobbies, and self-care. Higher scores indicate greater cognitive impairment [32]. Alzheimer’s is diagnosed by drawing blood to check for AD-related factors and judging dementia when CDR SB > 2.5 (CDR > 0.5) [33]. In addition, the Syndrome-Kurz Test (SKT) and 15-item Boston Naming Test (BNT) are commonly used to assess cognitive function in patients with AD. The Quality of Life-Alzheimer’s Disease (QoL-AD) scale is also used to assess QoL in patients with Alzheimer’s [34].

In addition to cognitive dysfunction, patients with AD experience physical functional problems such as balance, walking ability, and postural instability due to muscle atrophy, decreased muscle mobility, and decreased vestibular function [35]. These findings highlight the importance of assessing physical function. Muscular endurance is assessed using the 6-m walk test, and muscle strength is measured using the 5-times sit-to-stand and 30-s chair stand tests. Balance is assessed using the Tinetti Gait and Balance scale, a Modified Berger Balance Scale (BBS) test, and functional mobility is assessed using the timed up-and-go (TUG) test and gait speed. Fall-related fitness can be assessed by using the Senior Fitness Test Manual (SFT). These measures include upper extremity strength, lower extremity strength, total body endurance, upper extremity flexibility, lower extremity flexibility, and dynamic equilibrium [36].

### 4.2. Lewy Body Dementia

Lewy body dementia is a hallmark of PD and is characterized by a combination of gait, cognitive, and psychiatric symptoms, making the objective assessment of symptoms and signs difficult. However, clinical measures have been developed to standardize opinions among clinicians and to distinguish between symptoms and signs in new drug trials. The Hoehn-Yahr scale, published in 1967, is the most widely used test for PD. The scale divides Parkinson’s disease into five stages based on the severity of symptoms. Stage 1 is characterized by resting tremor or rigidity in one arm or leg. Stage 2 involves resting tremor or rigidity on both sides of the body. Stage 3 is marked by bilateral tremor. These symptoms become more severe with each subsequent stage; in stage 4, the person retains some ability to walk and stand but becomes incapacitated and has difficulty performing daily activities independently. The fifth stage is characterized by the inability to walk, stand, or perform anything while lying in bed [37].

The 10-Meter Walk Test (10 MWT), BBS, Functional Gait Assessment (FGA), and TUG are meaningful assessment tools. The 10 MWT is used to assess walking ability, while the BBS is a 14-item scale that includes static and dynamic balance ability and is considered a clinical balance scale. The FGA is used to assess dynamic balance function, including postural control during walking, with higher scores indicating better balance. Additionally, the FGA can be used to differentiate patients with mild and severe gait disorders. The TUG assesses gait and functional mobility, and its values correlate with the incidence of falls [38].

### 4.3. Frontotemporal Dementia

The average duration of survival from symptom onset in individuals with FTD is estimated to be 6.6 to 11.0 years [39]. After receiving a clinical diagnosis of FTD, the average survival time is estimated to range from three to four years [40]. These statistics highlight the considerable gap between symptom onset and formal diagnosis [41]. Consequently, the timely diagnosis and treatment of FTD can significantly influence the lifespan of affected individuals.

A comprehensive evaluation is necessary to make a precise diagnosis of FTD. This evaluation should include an assessment of the patient’s behavioral and cognitive history, behavioral changes, family history, neuropsychiatric examination, laboratory tests, and imaging studies. It is important to note that individuals with FTD commonly display symptoms such as anhedonia and emotional blunting, which may be erroneously attributed to major depression. Obsessive-compulsive disorder can be misdiagnosed as repetitive and impulsive behaviors, while bipolar disorder can be mistaken for delusions and euphoria [42].

The clinical diagnosis of FTD is based on the 2011 revision of the FTDC criteria. While the original FTDC was broadly designed for use in common dementias, this revision led to an assessment of the internal reliability of the new FTDC criteria for bvFTD, which were found to be more reliable than the Lund-Manchester criteria used for assessment in 1999 [43]. As a result of these initiatives, a new assessment tool for FTD was created in 2023. The Behavioral Dysfunction Questionnaire (BDQ) was designed to evaluate the frequency and severity of behavioral symptoms associated with frontotemporal dementia (FTD), as reported by caregivers. The questionnaire aims to assess the behavioral symptoms experienced by individuals with FTD. The BDQ evaluates 12 behavioral domains: depressed mood, agitation, loss of motivation, solicitation, aggression, aggressive behavior, repetitive behavior, excessive talking, changes in personal hygiene, loss of awareness, incompleteness, and obsession. It comprises 34 questions that assess the frequency and severity of behavioral symptoms on a 5-point scale. Caregivers provided their responses based on their observations of the patient’s behavior over the past four weeks. When using the BDQ to assess behavioral symptoms, it is important to consider the dynamics between the patient and caregiver, the caregiver’s perspective, and the accuracy of the diagnosis. The BDQ is a valuable tool for evaluating behavioral symptoms in individuals with FTD, and is recommended by the National Institutes of Health [44].

As there is limited research on outcome measures, specifically for individuals with FTD, we can rely only on the outcome measures discussed in a single case study. It should be noted that the generalizability of these measures to all individuals with FTD may be challenging, but they may be more accurately applied to individuals with cognitive deficits or younger age groups. In this case study, a range of functional measures was employed to assess the underlying factors contributing to balance impairment. These measures included gait speed, lower body strength, and assessments of static and dynamic stability [45].

The 8-Foot Walk Test was used to measure comfortable and fast walking speeds. This test has been shown to help predict overall function, fall risk, and mortality in older adults [46]. Lower body strength was assessed using the 30-Second Chair Stand Test [47]. Functional mobility is evaluated using the TUG test, which shows good inter- and intra-examiner reliability regardless of cognitive function level [48]. The Modified Berg Balance Scale (MBBS), Four-Square Step Test, and Fullerton Advanced Balance (FAB) scale are used to assess static and dynamic balance [49]. The MBBS is preferred over the Berg Balance Scale because of its better ability to predict falls in patients with dementia [50]. The FAB is administered to patients who scored high on the MBBS because it includes more challenging tasks such as jumping and reaction balance and has good validity [51].

### 4.4. Mild Cognitive Impairment

MCI can be diagnosed by obtaining information from individuals who have knowledge of the patient or from trained clinicians who observe the patient, where concerns regarding cognitive changes are reported and there is objective evidence of impairment in one or more cognitive domains including memory, executive function, attention, language, and visuospatial skills [28]. Individuals with MCI are often misdiagnosed with dementia due to cognitive impairment. However, it is important to note that a diagnosis of dementia requires evidence of significant impairment in social or occupational functioning. There are three clinical diagnostic criteria for MCI: the revised Mayo Clinical Criteria published in 2003; the NIA-AA Criteria published in 2011; and the DSM-5 Diagnostic Criteria published in 2013. These criteria may differ in terminology; however, their core criteria are the same [52]. According to the DSM-5, MCI is a mild neurocognitive disorder characterized by the need for greater effort and compensation for independent everyday functioning, although individuals are still able to maintain their independence [53]. The Mini-Mental State Examination (MMSE) and Montreal Cognitive Assessment (MoCA) are commonly used to diagnose MCI. However, it has been reported that the MMSE is not suitable for detecting clinical signs of MCI and dementia, which has led to the promotion of the MoCA [54]. The MoCA is a brief screening tool for MCI that was developed to overcome the limitations of the MMSE. It comprises seven subitems: visuospatial/executive function (5 points), naming (3 points), memory (5 points), attention (6 points), language (3 points), abstraction (2 points), and orientation (6 points). A score of 25 or less out of a maximum of 30 indicates MCI [55].

Cognitive impairment in older adults with MCI is associated with declines in executive function, mobility, and gait. The TUG test, a physical assessment tool that measures the walking speed of older adults, is commonly used in this population [56]. The TUG test measures mobility by timing the performance of rising from a seated position, walking 3 m, turning, walking back, and sitting down. The average time between two trials determines the TUG score [57]. The grip strength test is another physical function test that allows quick and convenient assessment of muscle strength. Grip-strength testing is simple, fast, reliable, and cost-effective [58]. Grip strength can be measured in absolute and relative terms. Absolute grip strength reflects the strength of the arms, legs, and core muscles and is influenced by body size [59]. In contrast, relative grip strength has been used in ergonomic research to adjust for body size, and previous studies have shown that it is associated with cardiovascular risk factors and metabolic disorders [60].

## 5. Therapeutic Interventions for Dementia

### 5.1. Alzheimer’s Disease

To date, there is no cure for Alzheimer’s disease, and the emphasis of treatment is placed on delaying symptoms through a combination of drug and nondrug treatments [30]. Currently, acetylcholinesterase (AChE) and glutamine receptor inhibitors are the most widely used drugs [61]. Although pharmacological treatments were initially prioritized in the early stages of dementia, interest in nondrug therapies has increased as side effects have been reported [12]. In addition, because drug treatment alone cannot completely halt the decline in cognitive and daily living skills in people with dementia, various non-drug therapies such as cognitive rehabilitation therapy are being utilized as additional treatments [61].

Physical activity (PA) interventions have shown promise as a supplement to current pharmacological treatments for cognitive symptoms in patients with AD. Specifically, aerobic exercise has been found to have positive effects on cognitive function and may slow the rate of decline in individuals with AD. This is achieved by enhancing neurotrophin levels, neurogenesis, and vascularization, while mediating neuroinflammation and inhibiting neuronal damage [62]. 

Exercise therapy may improve Aβ protein and BDNF, factors associated with AD, among non-drug therapies. Previous studies have shown that a combination of upper extremity large-muscle exercises, hand sensory stimulation, and small-muscle exercises can improve cognitive function in patients with AD [63]. As more areas of the cerebral cortex receive sensations from the hand than from any other part of the body, this cortical activity can be interpreted as a positive effect of cortical activity and interactions with other nervous systems [12]. 

Cognitive exercise is a necessary component of exercise therapy for individuals with dementia and is gaining increasing attention in the literature. These cognitive exercises involve self-awareness of movement directions, patterns, and clear intentions during PA. During PA, “intentional behavior” increases neural activation and improves cognitive impairment.

One exercise intervention study investigating methods to improve physical function in people with AD found that, in addition to aerobic exercise, resistance training and yoga were effective [63]. In particular, yoga can be beneficial for older adults with dementia as it is low in weight or cardiovascular load and easily accessible at home, making it a better choice for older adults with AD. In addition, yoga has been reported to be effective in reducing the concentration of Aβ protein in the blood.

The various yoga poses have been found to increase cerebral blood flow when compared to other exercises. This increased cerebral blood flow can reduce the amount of amyloid precursor protein debris and Aβ proteins, potentially improving the body’s ability to break them down [12]. The accumulation of Aβ and tau proteins leads to Aβ plaque formation and tau neurofibrillary tangles which interfere with the normal functioning of neurons, most notably by reducing the expression of choline acetyltransferase, leading to the loss of certain neuronal subtypes. This enzyme blocks acetylcholine production, resulting in loss of normal brain function. However, practicing yoga releases several neurotransmitters, including serotonin, dopamine, and histamine, which interact with serotonin to increase the expression of choline acetyltransferase. These activate serotonin receptors to inhibit Aβ oligomer formation or inhibit nitric oxide synthesis. This could be considered a mechanism underlying the observed neuroprotective effects [64].

### 5.2. Lewy Body Dementia

Lewy body dementia is primarily treated pharmacologically using a combination of acetylcholinesterase inhibitors, antipsychotics, and dopamine agonists [65]. Acetylcholinesterase inhibitors, such as donepezil (also known as carserine), Galantamine, and Rivastigmine increase acetylcholine levels in the brain, thereby alleviating cognitive decline [66]. Antipsychotics such as quetiapine, risperidone, and olanzapine are primarily used to improve cognitive symptoms of dementia [67]. Furthermore, dopamine agonists, such as pramipexole and ropinirole, are used to manage movement disorders or Parkinson’s symptoms that occur in LBD by stimulating dopamine receptors to improve motor function [65]. It is important to maintain objectivity and avoid subjective evaluations when discussing medical treatments. 

An important consideration when prescribing these drugs is that they can be affected by the patient’s environment and induce late side effects, including memory problems, digestive disorders, movement disorders, confusion, sleep problems, neurological problems, and increased risk of premature death [65]. Non-drug treatments consist of occupational interventions to minimize dysfunction in the home environment, or physical therapy to improve gait. Various exercise modalities can improve cognitive and functional outcomes in patients with dementia, including gait speed, duration, and multidomain cognition [68]. They can also improve the function of patients with mild cognitive impairment by improving their gait duration, mobility, and disability [69]. In addition to these medications, physical therapeutic options are also available. Common exercise treatments for LBD include the following: First, strength-building exercises are recommended, targeting the abdominal, leg, and arm muscles and strengthening weakened muscles [70]. Second, stretching and joint range-of-motion exercises should be performed to improve flexibility. 

Balance and gait training improves balance and gait, which gradually weaken in patients with LBD. Such exercises may include standing on one foot, which strengthens ankle muscles, and improving gait patterns. Programs may also include exercises to improve patients’ cognitive abilities, because LBD can cause cognitive problems [71]. These exercises may involve remembering and following movements, moving through mazes, and improving intuitive movement patterns. Finally, training for activities of daily living involves assisting patients in performing daily tasks independently by practicing movements in real-world settings to maintain the ability to perform the movements required for daily life [72].

Recent studies have shown that tango dancing can be implemented as an effective intervention in patients with LBD and Parkinson’s symptoms. For example, Foster et al. found that repetitive motor learning through tango dancing improved functional outcomes [73]. Another study found that tango dance therapy led to significant improvements in patients with both LBD and PD [74]. These findings suggest that exercise with music can have a positive effect on patients with LBD and that music can increase patients’ motivation and interest and enhance the effectiveness of exercise. Exercises with music, such as tango dancing, can be considered as an alternative treatment for people with LBD that can provide psychological, cognitive, and motor benefits [74].

Tango dancing can have beneficial effects not only athletically but also psychologically and cognitively. Tango dancing improves motor coordination and adaptability and can also help improve cognition. Particularly, the process of learning and memorizing steps and different postures through tango dancing promotes cognitive performance and memory [75].

### 5.3. Frontotemporal Dementia

Currently, the U.S. Food and Drug Administration has not approved any specific medications or treatments solely for FTD. Nevertheless, several pharmacological approaches have been explored to address the behavioral, motor, and cognitive symptoms associated with FTD. These strategies primarily involve replacement and modulation of neurotransmitters. Examples of such medications include selective serotonin reuptake inhibitors, atypical antipsychotics, acetylcholinesterase inhibitors, and glutamate NMDA receptor antagonists [22]. Medication plays an important role in the management of patients with FTD. However, it cannot completely eliminate negative behavioral symptoms. Adequate management of the behavioral symptoms of FTD requires a combination of medication and behavioral, physical, and environmental mental modification techniques [76].

Currently, there is limited research on the effects of exercise in individuals with FTD. However, a meta-analysis of studies focusing on exercise interventions in individuals with mild dementia revealed significant positive effects, ranging from moderate to large, on the activities of daily living. Additionally, the analysis demonstrated moderate positive effects on mood when cognitive and physical interventions were combined [77].

In the context of FTD, psychobehavioral issues manifest at an early stage, while motor and cognitive impairments progress over time. Therefore, it may be beneficial to adjust exercise approaches for individuals in the early stages of dementia. Additionally, the primary goal of treating advanced dementia is to alleviate behaviors that may indicate discomfort and psychological distress. To tackle this issue, music therapy is used as a strategy that employs rhythms and music to alleviate symptoms and promote emotional regulation [78]. Engaging in rhythmic singing has been shown to activate various brain regions, including the bilateral supplementary motor cortex, with notable emphasis on the anterior cingulate cortex in the left hemisphere and the basal ganglia in the right hemisphere [79]. Langhammer et al. (2019) conducted a study on eight patients diagnosed with AD and vascular dementia exhibiting frontotemporal symptoms. The study implemented a combination of individualized music therapy and increased physical activity over the course of eight weeks. The findings revealed that the intervention reduced anxiety, restlessness, irritability, and aggression in the participants [80]. Hence, it could be valuable for therapists and families to explore the use of a combination of physical activity and musical elements to address early behavioral and emotional alterations in individuals with frontotemporal lobe degeneration.

Currently, studies specifically investigating exercise interventions in individuals with FTD are lacking. Therefore, research focusing on emotion regulation, which is challenging for this patient group, may provide valuable insights. One study examining the effects of aerobic exercise and mindfulness-based yoga on emotion regulation demonstrated significant improvements over aerobic exercise alone. The involvement of the medial prefrontal cortex in the dopaminergic circuits could explain this phenomenon. Aerobic exercise can continuously stimulate these circuits, potentially enhancing resilience to negative emotional influences [81]. Additionally, a unified protocol may be beneficial for individuals struggling with difficulties in emotion regulation. This study proposes the adoption of a more comprehensive approach to disease categorization, moving away from excessive categorization and embracing a ”transdiagnostic approach”. Embracing a “transdiagnostic approach” involves the recognition of shared features among disorders, rather than focusing solely on minor variations in symptoms. Through the implementation of a unified protocol, specific aspects such as thoughts, bodily sensations, and behaviors can be examined thoroughly. Such protocols emphasize the identification of maladaptive emotion regulation strategies that individuals have developed over time within these domains and aim to teach them more adaptive emotion regulation skills. By enhancing patients’ understanding of their emotions, we can assist them in better regulating their emotions, which is particularly beneficial for those who struggle with emotion regulation difficulties [82]. Therefore, based on the results of previous studies, we recommend implementing aerobic exercise strategies for patients with FTD who have difficulty regulating their emotions. However, by applying methods such as a unified protocol beforehand, we may be able to develop more efficient and novel exercise protocols.

### 5.4. Mild Cognitive Impairment

Physical exercise is recognized as an effective nonpharmacological intervention for enhancing cognitive function and delaying cognitive decline in patients with MCI [83]. Other studies have demonstrated that physical exercise reduces cytokine levels and improves peripheral concentrations of neurotrophic factors, thereby delaying cognitive decline [84]. In particular, aerobic exercise has shown a greater efficacy in mitigating cognitive decline than other forms of exercise, as supported by numerous studies [85]. According to Öhman, physical activity prevents cognitive decline and dementia in older adults, and protects against early-stage damage in cognitively healthy individuals. Research has shown that regular aerobic exercise can lead to improvements in physical and cognitive functions by inducing changes in cardiovascular and mood-related factors [28]. Aerobic exercise increases the serum levels of BDNF, enhances hippocampal size, and improves cerebral blood flow and oxygen supply to the brain, thereby enhancing neurotransmitter availability and efficiency [85]. Additionally, aerobic exercise has been found to promote cognitive function.

Baduanjin, Pilates, and yoga are functional exercises that focus on body movements and enhance cognitive function. According to recent studies, Thakkar et al. discovered that when individuals perceive their body movements, brain cells grow rapidly and the neural system becomes better connected throughout the body [86]. Zheng et al. investigated the effect of Baduanjin exercises on cognitive function in patients with stroke. This study compared the cognitive function, specific domains (memory, processing speed, executive function, attention, and visuospatial abilities), and daily activities. The Baduanjin exercises were associated with significant differences in cognitive function, executive function, memory, attention, and daily activities. This confirms that Baduanjin exercises can improve not only physical aspects through simple movements and sequences, but also attention and focus [87]. Pilates is another type of physical exercise that allows individuals to concentrate on body movements. According to a related study, Pilates significantly enhances cognitive function, agility, dynamic balance, and overall functional status. These findings support the classification of Pilates as a functional exercise [88]. Yoga focuses on movement within the body space and enhances proprioceptive awareness and consciousness. Yoga engages the muscles through the maintenance of specific postures, thereby promoting a state of concentration. A similar phenomenon was observed during meditation. According to Thakkar et al., meditation increased blood flow to the relevant areas of the brain and elevated oxyhemoglobin levels in the prefrontal cortex, resulting in improved cognitive abilities [86].

In conclusion, both aerobic and functional exercises that focus on bodily movements, such as yoga, have been shown to contribute to improvements in cognitive ability. In particular, yoga has been shown to significantly enhance cognitive function. These findings suggest that physical exercise not only has physical benefits but also improves attention and concentration through movement patterns and sequences, which are expected to have meaningful effects on individuals with MCI (Table 1). 

## 6. Conclusions

### 6.1. Implications

This paper provides important implications and practical contributions in the field of dementia care. It highlights the significance of individualized non-pharmacological interventions that are tailored to each specific type of dementia, such as Alzheimer’s Disease, Lewy Body Dementia, Frontotemporal Dementia, and Mild Cognitive Impairment. The paper discusses the effectiveness of physical exercises and activities, such as yoga, tango dancing, and music therapy, in enhancing cognitive function and quality of life in individuals with dementia. The benefits of these therapies are highlighted, including their cost-effectiveness, fewer side effects compared to medication, and potential for easy integration into daily routines. The importance of personalized and diversified treatment strategies beyond traditional pharmacological methods is underlined by the predicted increase in dementia cases. The research indicates a paradigm shift towards more holistic and individualized care in dementia treatment, which could lead to better management and outcomes for patients.

### 6.2. Limitations and Future Research

While this theoretical review provides a comprehensive analysis of non-pharmacological interventions for dementia and their potential benefits, it may be limited by its potential for bias, lack of systematic methodology, and reduced generalizability compared to systematic reviews. As a future approach, a systematic review will allow for a more rigorous and comprehensive synthesis of existing research, involving a methodical collection, appraisal, and summary of all relevant studies on the topic. This approach will enable us to provide stronger evidence and more definitive conclusions about the effectiveness of different non-pharmacological interventions for each type of dementia.

### 6.3. Summary and Conclusion

This comprehensive study has provided a detailed overview of the most prevalent types of dementia: Alzheimer’s Disease, Lewy Body Dementia (LBD), Frontotemporal Dementia (FTD), and Mild Cognitive Impairment (MCI). Each form of dementia presents unique challenges in both diagnosis and management, necessitating an individualized approach. 

In Alzheimer’s Disease, the most common form of dementia, the focus is on identifying cognitive decline, memory loss, and language difficulties. The study underscores the effectiveness of non-pharmacological interventions, such as aerobic exercises and yoga, which are instrumental in managing symptoms. Diagnostic methods primarily include cognitive testing and physical function assessments, reflecting the disease’s characteristic cognitive and motor symptomatology. Lewy Body Dementia is distinguished by a combination of cognitive impairments and Parkinson’s disease-like symptoms. Our findings highlight the importance of using Parkinson’s disease-related scales, such as the Hoehn-Yahr scale, for diagnostic purposes. Non-pharmacological treatments like physical therapy and tango dancing show promise in improving patient outcomes, underscoring the need for multifaceted treatment approaches. Frontotemporal Dementia, characterized by notable changes in behavior, executive functions, and language, requires a nuanced approach to diagnosis and management. The Behavioral Dysfunction Questionnaire emerged as a key tool for diagnosing FTD, with music therapy and exercise interventions showing potential in treatment. Mild Cognitive Impairment, a precursor to more severe forms of dementia, necessitates early and accurate diagnosis through cognitive assessments such as the MMSE and MoCA. Our study emphasizes the role of physical exercises, particularly aerobic activities, in delaying or mitigating the progression to more severe dementia forms.

In conclusion, this study highlights the necessity of tailored diagnostic and therapeutic strategies for dementia. The adoption of non-pharmacological treatments, including physical and cognitive activities, is crucial in managing dementia symptoms and enhancing patient quality of life. Moreover, this research advocates for the importance of early and precise physiotherapeutic diagnosis in dementia care, paving the way for effective and personalized management strategies. 

## Figures and Tables

**Table 1:** Symptoms and therapeutic interventions for different types of dementia.

Type	Symptoms	Therapeutic Interventions
Alzheimer’s Disease	Progressive cognitive decline Memory loss Difficulties with language Decreased physical function	Aerobic exercise to enhance neurotrophin levels and slow cognitive decline Resistance exercise to improve brain-derived neurotrophic factor (BDNF) Yoga to potentially reduce amyloid-beta protein concentration and improve cerebral blood flow Cognitive exercises to improve self-awareness and movement patterns
Lewy Body Dementia	Impaired memory and attention Hallucinations Parkinson’s disease-like symptoms (e.g., tremors, rigidity) Sleep disturbances Episodic delusions	Physical therapy to improve gait and balance Strength-building and flexibility exercises Activities to enhance cognitive abilities and functional mobility Tango dancing as a form of exercise therapy to improve coordination and cognition.
Frontotemporal Dementia	Decline in behavioral and executive functions Language impairment Personality changes Potential motor neuron disease overlap	Exercise interventions focusing on mood and activities of daily living Music therapy to mitigate symptoms and promote emotional regulation Potentially aerobic exercise for emotion regulation improvements Application of a Unified Protocol to address emotion regulation difficulties
Mild Cognitive Impairment	Cognitive decline greater than expected for age and education level but not significantly interfering with daily life Difficulties in memory, executive function, attention, language, and visuospatial skills	Regular aerobic exercise to prevent cognitive decline and dementia Functional exercises like Baduanjin, Pilates, and yoga to improve cognitive function and enhance proprioceptive awareness Meditation and focused movement to improve cognitive abilities

## Data Availability

The data presented in this study are available upon request from the corresponding author.
